# Dose/Volume histogram patterns in Salivary Gland subvolumes influence xerostomia injury and recovery

**DOI:** 10.1038/s41598-019-40228-y

**Published:** 2019-03-05

**Authors:** Peijin Han, Pranav Lakshminarayanan, Wei Jiang, Ilya Shpitser, Xuan Hui, Sang Ho Lee, Zhi Cheng, Yue Guo, Russell H. Taylor, Sauleh A. Siddiqui, Michael Bowers, Khadija Sheikh, Ana Kiess, Brandi R. Page, Junghoon Lee, Harry Quon, Todd R. McNutt

**Affiliations:** 1 Department of Radiation Oncology and Molecular Radiation Sciences, Johns Hopkins University, Baltimore, MD USA; 2 Department of Civil Engineering, Johns Hopkins University, Baltimore, MD USA; 3 Department of Computer Science, Johns Hopkins University, Baltimore, MD USA; 4 Department of Public Health Sciences, University of Chicago, Chicago, IL USA; 5 Department of Epidemiology, Johns Hopkins Bloomberg School of Public Health, Baltimore, MD USA

**Keywords:** Outcomes research, Head and neck cancer

## Abstract

Xerostomia is a common consequence of radiotherapy in head and neck cancer. The objective was to compare the regional radiation dose distribution in patients that developed xerostomia within 6 months of radiotherapy and those recovered from xerostomia within 18 months post-radiotherapy. We developed a feature generation pipeline to extract dose volume histogram features from geometrically defined ipsilateral/contralateral parotid glands, submandibular glands, and oral cavity surrogates for each patient. Permutation tests with multiple comparisons were performed to assess the dose difference between injury vs. non-injury and recovery vs. non-recovery. Ridge logistic regression models were applied to predict injury and recovery using clinical features along with dose features (D10-D90) of the subvolumes extracted from oral cavity and salivary gland contours + 3 mm peripheral shell. Model performances were assessed by the area under the receiver operating characteristic curve (AUC) using nested cross-validation. We found that different regional dose/volume metrics patterns exist for injury vs. recovery. Compared to injury, recovery has increased importance to the subvolumes receiving lower dose. Within the subvolumes, injury tends to have increased importance towards D10 from D90. This suggests that different threshold for xerostomia injury and recovery. Injury is induced by the subvolumes receiving higher dose, and the ability to recover can be preserved by further reducing the dose to subvolumes receiving lower dose.

## Introduction

Precision medicine takes into account variability in a patient’s disease state, to improve healthcare outcomes by personalizing individual’s treatment^1^. In radiation therapy (RT), a precision medicine paradigm requires the ability to characterize important sources of heterogeneity that are not only present in disease states, but in the patient’s treatment. It is believed that predicting treatment toxicities associated with each individual’s treatment parameters is important for further clinical decision support, such as optimizing treatment plans. One such toxicity is xerostomia (dry mouth), which is one of the most commonly reported RT-induced adverse events among head and neck cancer (HNC) patients^2,3^.

Clinically significant xerostomia is defined as grade ≥2 using the NCI-CTCAE criteria^4^. Studies have indicated that the pathophysiology of xerostomia may be different from early phase (within 2 months post-RT) to late phase (8 months to 1 year): the early phase may be attributed to apoptosis of acinar cells, granular shrinkage or membrane damage-induced dysfunction^5–7^, while the late phase may be attributed to an inability of replacing the apoptotic acinar cells, resulting from radiation-induced reduction of the stem/progenitor cells^3,8,9^. It was previously believed that radiation-induced xerostomia is an irreversible, life-long problem^5^. However, some clinical studies have demonstrated that patients with clinically significant xerostomia may recover some salivary function. By analyzing the flow rate and components of stimulated and unstimulated saliva after RT, Richard *et al*.^10^ found that compared to patients who had no xerostomia, patients who had xerostomia (LENT-SOMA grade ≥2) showed significantly decreased flow rate of stimulated and unstimulated saliva at 3–6 months after intensity-modulated radiotherapy (IMRT) but not at more than 12 months post-RT.

Radiation dose to the salivary glands has been widely studied for the risk of xerostomia. Mean dose to the whole ipsilateral/contralateral parotid gland (iPG, cPG) affects xerostomia incidence, but not the recovery of saliva function^11,12^. In terms of RT-induced salivary gland dysfunction, the radiosensitivity of the PG is not homogeneous^13–17^. There is no consensus on the most radiosensitive region in the PGs: previous work has shown the most radiosensitivity regions for xerostomia incidence includes the cranial aspects^13^, dorsal aspects adjacent to mandible^14^, caudal-lateral aspects^15^, caudal-anterior aspects^16^, and the superficial lobe^17^. Although advanced machine learning models have improved prediction using high-dimensional reduction methods^15,18^, the translatability of these study results for clinical dose-volume histogram (DVH) based treatment planning might be limited.

Despite the clinical significance of xerostomia, our ability to prevent it remains limited due to the lack of comprehensive dosimetric analysis of normal structures involved in salivary dysfunction. Thus, to better understand the regional dosimetry of normal structures involved in xerostomia, we sought to investigate the different influence of DVH regional dose/volume metrics patterns between xerostomia injury and recovery. We incorporated segmented RT structures as PG, submandibular glands (SMG) and oral cavities surrogates (OC). We hypothesized that the regional dose/volume metrics patterns to the salivary glands are different between injury and recovery, and can be evaluated with dosimetric features of partitioned subvolumes. Sustained xerostomia after RT might be reduced by the selective sparing of subvolumes of the PG or other salivary gland tissues.

## Methods and Materials

### Study design and data collection

Patient assessments data such as oncologic toxicity outcomes and symptom management have been collected prospectively at the point of care using an online data capturing tool coupled with the departmental database: Oncospace^TM^ ^19,20^. Follow-up schedule is based on NCCN guideline^21^, which consists of a 6 weeks follow-up, followed by appointments every 3 months for 2 years, every 6 months through 3–5 years and yearly thereafter. Dosimetric and volumetric data, including for example, region of interest (ROI) contours, shape relationships, and dose-volume histogram (DVH) curves have been captured using the Oncospace^TM^. This retrospective study was approved by Institutional Review Board (IRBX) of Johns Hopkins Medicine. Written consent was not obtained from the study participants because the retrospective data used encrypted identification of the individuals. This study was given a formal waiver for the need for consent by the Institutional Review Board. All methods were performed in accordance with the HIPPA regulations.

### Study population

The primary outcome was the physician-assessed xerostomia grade based on following scoring criteria. 0: No xerostomia. 1: Symptomatic (e.g., dry or thick saliva) without significant dietary alteration. 2: Moderate symptoms; oral intake alterations (e.g., copious water, other lubricants, diet limited to purees and/or soft, moist foods). 3: Inability to adequately aliment orally; tube feeding or TPN indicated. The scoring is based on NCI-CTCAE 4.0, however in our clinic, patients’ unstimulated saliva flow rate is not directly measured as often practiced when this criteria is used in cooperative group trials. From Oncospace^TM^, we observed that clinical significant xerostomia (xerostomia grade ≥2) usually occurs within 6 months of RT for HNC patients, and some patients sustained long-term damage with xerostomia (non-recovery) assessed at and over 18 months of post-RT. Thus, in this study, “injury” was defined as any incidence of grade ≥2 xerostomia within 6 months of RT, and “recovery” was defined as injury, followed by a reduction of xerostomia score to <2 within 18 months post-treatment.

**Figure 1:**
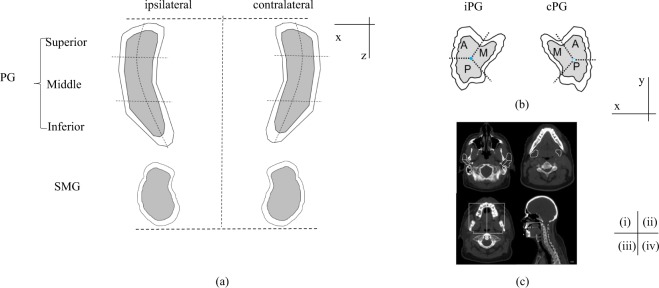
Schematic of contour segmentation. Segmentation of parotid gland (PG) and submandibular gland (SMG) are shown in coronal view (**a**) and transverse view (**b**). A shell of 3 mm expansion of parotid gland was created. M: medial; A: anterior; P: posterior. Figure 2(c) shows the contours of bilateral PG (i), SMG (ii) and OC (iii: transverse view and iv: sagittal view) in image set, delineated in white.

We created a radio-morphologic feature generation pipeline to extract DVH features from derived subvolumes of the salivary glands^22^. First, we mapped laterality for each patient to ipsilateral and contralateral based on the primary tumor location. Second, an expansion of 3 mm was applied to the contours of PGs and SMGs to account for setup error and a high dose gradient at the edge of them. Next, each PG was partitioned into three radial sectors in the axial plane with the equal angle (medial, anterior and posterior), and three superior-inferior sections with the equal distance (superior, middle, inferior). Finally, as oral cavity was not consistently contoured, a surrogate was defined as the area outside of PG, SMG and mandible, bounded superiorly by lower 2/3 of the PG, inferiorly by the inferior SMG, and anteriorly and posteriorly by the mandible. This OC was divided along the medial axis to identify ipsilateral/contralateral halves. This set of transformation resulted in 22 derived subvolumes, including ipsilateral/contralateral PG (iPG, cPG), ipsilateral/contralateral SMG (iSMG, cSMG), ipsilateral/contralateral OC (iOC, cOC) (Fig. 1). For each derived subvolume, DVH features were calculated in 10% volume increments from D10 (dose covering 10% of subvolume) to D90 (dose covering 90% of subvolume), producing 198 dosimetric features per patient. The distribution of the volume for each subvolume in the PGs is shown in Supplement Table 1.

**Figure 2:**
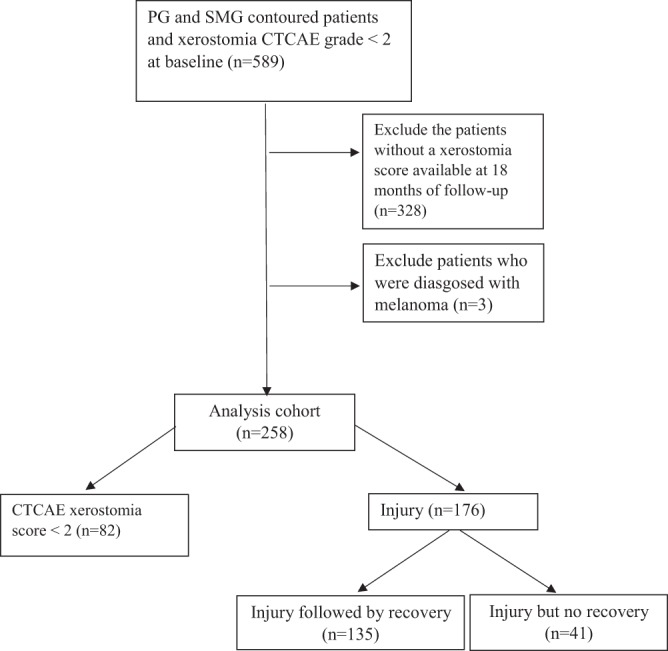
Flowchart of patient selection, with exclusion criteria.

HNC patients with both PGs and SMGs contoured and who underwent primary RT between 2007 and 2017, except for those who had xerostomia injury before RT (n = 589) were included in this study. Patients without a xerostomia score available at 18 months of follow-up (n = 328) and patients who had melanoma (n = 3) were excluded (Fig. 2). This resulted in 258 HNC patients. Among the identified 258 HNC patients, 82 patients had no xerostomia, 176 patients developed clinical significant xerostomia (CTCAE grade ≥2) within 6 months of RT, and 135 of them recovered before 18 months post-RT (Fig. 2).

### Statistical analysis

To test if any significant dose difference exists between groups, multiple comparisons permutation tests were performed and replicated on 1000 random samples^23,24^ on dose features for injury and recovery outcomes separately. Significance was determined by a one-sided adjusted p-value on 5% level, which is the proportion of the higher normalized maximum in the resampled sets than the observed sample. The dose features that were statistically different between injury vs. non-injury (adjusted p-value <0.05) and recovery vs. non-recovery (adjusted p-value < 0.05) were considered important features.

Logistic regression with ridge regularization was used to build the injury and recovery prediction models. Ridge logistic regression regularizes the coefficients by assigning larger weights to more important features, thus making it preferred for learning relative importance for highly correlated dosimetric features^25^. The feature’s weight (β), indicating how a single unit increase of it influences a change in the “odds” of injury and recovery separately in a log scale, was learned by the models combined with regularization hyperparameter tuning using 5-fold cross-validation. β > 0 suggests that the an increase of the feature is correlated with the increased probability of the dependent variable (injury or recovery), while β < 0 suggests that an decrease of the feature is correlated with the increased probability of the dependent variable. The following clinical factors were also included in both injury and recovery models: age at start of RT (≤65, >65), gender (male, female), race (white, African American and other), had ever received chemotherapy (yes, no), tumor site (nasopharynx, oral cavity, oropharynx, hypopharynx and larynx), volumes of salivary glands pre-RT (iPG, cPG, iSMG, cSMG) and volumes of oral cavity surrogates (iOC and cOC). Dose feature importance was normalized to be in the range of [−100, 100] and was further visualized.

Given the sample sizes (258 for injury and 176 for recovery), the generalization error estimation on a single randomly separated test dataset was likely to be biased^26^. Nested cross-validation (Nested CV)^27^ was thus used to assess the model performance. Nested CV effectively uses a series of training/validation/test set splits. In the inner loop, the performance is approximately maximized by fitting a model to each training set, and then directly maximized in selecting regularization hyperparameters over the validation set. In the outer loop, generalization error is estimated by averaging test set performances over several dataset splits, thus avoiding overfitting. Cross-validation was performed with 5-folds in the inner loop and 5-folds in the outer loop.

The ridge logistic regression models with Nested CV were developed with DVH features extracted from the subvolumes (segmented PG, SMG and OC) for contours + 3 mm expansion shells to predict injury and recovery of xerostomia separately. The 3 mm expansion was used to account for a dose gradient between the organ at risk and the surrounding tissues in the “extra-organ space” and to account for organ at risk motion and set-up uncertainty during treatment. For both the injury and recovery outcomes, the area under the receiver operating curve (AUC), sensitivity, and specificity were used to assess the performance of the models; standard deviations for AUC, sensitivity, and specificity were determined from re-sampling of training/validation/test datasets with 50 iterations.

All the statistical analyses were done using STATA (StataCorp. 2017. Stata Statistical Software: Release 15. College Station, TX: StataCorp LLC) and R Project for Statistical Computing (Version 3.2.3, Vienna, Austria).

## Results

As summarized in Table 1, 82 patients presented with no xerostomia (group i), 135 patients presented with injury followed by recovery (group ii), and 41 patients presented with injury but never recovered (group iii). No obvious age differences between the three groups were observed. Patients with injury (ii and iii) were likely to have tumor sites of “oral cavity” and “oropharynx”, have stage T2 and above, lymph nodes involved (N1 and above), tumor metastasis (M1), and chemotherapy compared to patients with no xerostomia (i). Compared to patients with injury followed by recovery (ii), patients with injury but no recovery (iii) were likely to have tumor site at “oral cavity” and “oropharynx”, have higher T stage and M stage, and smaller pre-treatment salivary gland volumes (Table 1).

**Table 1:** Patients Characteristics by Xerostomia Injury and Recovery.

	Type of Patients		
	No xerostomia (i: N = 82)	Injury and Recovery (ii: N = 135)	Injury but No recovery (iii: N = 41)
**Age >65 years** ^*^	26 (31.7)	35 (25.9)	11 (26.8)
**Race** ^*^			
Caucasian	61 (75)	109 (78)	36 (83)
African American	19 (23)	23 (17)	4 (10)
Other	2 (2)	3 (2)	1 (2)
**Gender** ^*^			
Female	24 (29)	21 (16)	11 (27)
Male	58 (71)	114 (84)	30 (73)
**Tumor site** ^*^			
Hypopharynx	2 (2)	2 (1)	2 (5)
Larynx	15 (18)	20 (15)	0 (0)
Nasopharynx	3 (4)	7 (5)	0 (0)
Oral Cavity	11 (13)	43 (32)	12 (29)
Oropharynx	27 (33)	51 (38)	22 (54)
Others	24 (29)	12 (9)	5 (12)
**T stage** ^**^			
0 and 1	25 (30)	38 (28)	5 (12)
2 and above	43 (52)	88 (65)	30 (73)
missing	14 (17)	9 (7)	6 (14)
**N stage** ^**^			
0	31 (38)	36 (27)	8 (20)
1 and 2	37 (45)	88 (65)	26 (63)
missing	14 (17)	11 (8)	7 (17)
M stage**			
M0	63 (77)	121 (90)	33 (80)
M1	1 (1)	2 (1)	3 (7)
missing	18 (22)	12 (9)	5 (12)
Chemotherapy*			
Yes	49 (60)	99 (73)	27 (66)
No	33 (40)	36 (27)	14 (34)
**Volume – cc** ^***^ **(mean ± sd)**			
iPG	57.9 ± 18.6	60.4 ± 18.6	53.6 ± 18.7
cPG	58.6 ± 17.8	60.7 ± 18.6	54.6 ± 17.5
iSMG	19.9 ± 7.2	21.3 ± 11.2	18.7 ± 5.4
cSMG	21.0 ± 6.1	21.5 ± 11.3	18.9 ± 5.2
iOC	178.1 ± 42.7	180.3 ± 40.01	177.1 ± 38.9
cOC	176.6 ± 41.7	178.9 ± 40.1	177.2 ± 40.0

*Categorical variables were displayed as count and percentage.

**American Joint Committee on Cancer (AJCC) staging 7.0^th^ edition. For T stage, 0 includes T0 and Tis; 4 includes T4a and T4b. For N stage, 2 includes N2a, N2b and N2c.

***Continuous variables were displayed as mean ± SD. Volume includes contours +3 mm shell.

**Figure 3:**
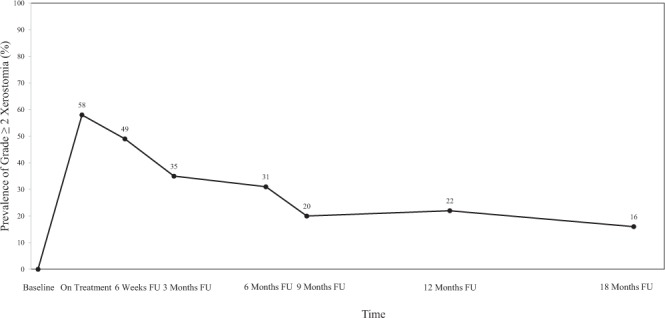
The prevalence plot for CTCAE xerostomia grade ≥2 during follow-up. FU: follow-up.

Among 258 HNC patients identified, 58% of them developed grade ≥2 xerostomia during on-treatment visits (OTV) (Fig. 3). The prevalence of xerostomia decreased thereafter, only 16% of the patients still had grade ≥2 xerostomia at 18 months post-treatment, indicating the recovery process (Fig. 3).

**Figure 4:**
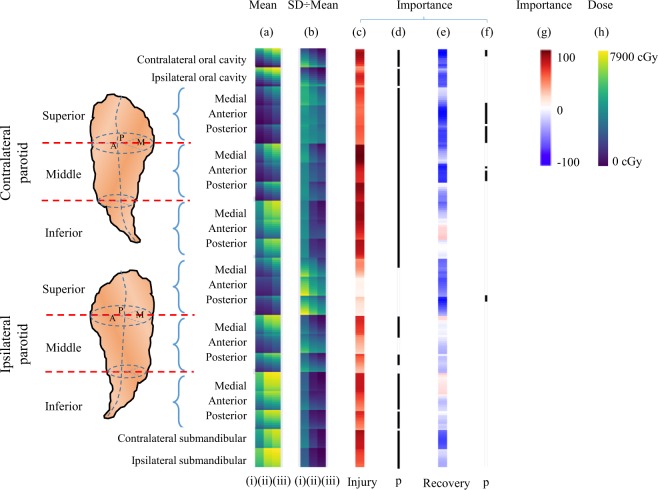
Dose distribution patterns for patients with (i) no xerostomia, (ii) injury followed by recovery, and (iii) injury but no recovery: (**a**) mean dose features (range 0–7900 cGy), (**b**) coefficient of variation for the following subvolumes: cOC, iOC, cPG, iPG, cSMG and iSMG. The feature importance resulting from the prediction models is shown for the (**c**) injury model (darker red indicates that an increase of dose is more influential to injury risk) and (**e**) recovery model (darker blue indicates a decrease of dose is more influential for improving the recovery chance, whereas red indicates an increase of dose is influential for improving the recovery chance). The black bars indicate significant differences (p < 0.05) for features between (**d**) injury (ii and iii) and no xerostomia (i), and (**f**) injury followed by recovery (ii) and non-recovery groups (iii). The relative importance (normalized to [−100, 100]) is shown in (**g**), and radiation dose range is shown in (**h**). Both PGs are segmented into the superior, middle and inferior portion, which are further segmented to medial, anterior and posterior sectors.

Figure 4 Shows for each structure, (contralateral oral cavity, ipsilateral oral cavity, contralateral superior parotid gland, contralateral middle parotid gland, contralateral inferior parotid gland, ipsilateral superior parotid gland, ipsilateral middle parotid gland, ipsilateral inferior parotid gland, contralateral submandibular gland, and ipsilateral submandibular gland), the dose to a certain percentage of volume in 10% increments. Figure 4a demonstrates the mean for each of the three groups identified, and Fig. 4b identifies the coefficient of variation (i.e. standard deviation divided by mean). The parotid glands are further divided to indicate the medial, anterior, and posterior regions. Individual dose features for each patient with no xerostomia, injury followed by recovery, and injury but no recovery are shown in Supplement Fig. 1.

Visually (Fig. 4a), it is apparent that the patients without xerostomia (i) received lower overall dose compared to those patients who had xerostomia and then recovered (ii), and to those patients who did not recover from xerostomia (iii). This is indicated by the cooler colors (greater regions of blue and green in the Figure h). It is also visually apparent looking at the ratio of the standard deviation to the mean (Fig. 4b) that patients who did not have xerostomia (i) received a greater range of dose, compared to patients who had xerostomia at same percent of volume (ii and iii).

To demonstrate which dose volumes are different between the three groups, a permutation test with multiple comparisons was performed. The black bars identify the important dose volume levels. Briefly for injury, the figure shows the following: (1) dose to the both oral cavities, both submandibular glands, contralateral parotid gland, middle and inferior portion of ipsilateralparotid gland is important, and (2) dose to the superior portion of the ipsilateral gland is not as important (Fig. 4d). For recovery, however, the black bars indicate that the superior portion of both parotid glands and contralateral oral cavity is important (Fig. 4f).

To quantify the importance of the regions, ridge logistic regression models for injury and recovery were performed. Darker red indicates greater importance of increasing dose, while darker blue indicates greater importance of decreasing dose, and white indicates no importance. For injury, dose to cOC, middle and inferior cPG demonstrate higher relative importance, while dose to superior iPG shows little importance. Among the majority of the subvolumes, high dose to lower volume features (e.g., D10-D40) is more important than the low-dose bath (e.g., D70-D90). These subvolumes are cOC, superior-medial cPG, middle-anterior cPG, middle-posterior cPG, inferior-anterior cPG, inferior-posterior cPG, superior-posterior iPG, middle iPG, inferior-anterior iPG, inferior-posterior iPG, and both SMGs.

In contrast, for recovery, dose to cOC, superior-anterior cPG, superior-posterior cPG, middle-anterior cPG and superior-posterior iPG demonstrate higher relative importance for reducing chance of recovery (Fig. 4e, darker blue). Dose to other subvolumes shows low to moderate importance. Increasing dose to superior-medial cPG, middle-medial cPG, middle-posterior cPG, inferior-anterior cPG, inferior-posterior cPG, middle iPG, and inferior-medial iPG appears to improve the chance of recovery, however, the relative importance values are low (Fig. 4e, red). The coefficients of DVH features derived from the model are summarized in Supplement Table 2.

The prediction models yielded stable performances and calibration. For injury, the Nested CV AUCs, sensitivities, and specificities were 0.78 ± 0.0009, 0.77 ± 0.003 and 0.74 ± 0.008 (Table 2). For recovery, the Nested CV AUCs, sensitivities, and specificities were 0.70 ± 0.002, 0.71 ± 0.02 and 0.67 ± 0.02 (Table 2).

**Table 2:** Nested Cross-Validation AUC for Xerostomia Injury and Recovery.

Model	AUC ± SD	Senstivity ± SD	Specificity ± SD
Injury	0.78 ± 0.0009	0.77 ± 0.003	0.74 ± 0.008
Recovery	0.70 ± 0.002	0.71 ± 0.02	0.67 ± 0.02

## Discussion

To better understand the influence of spatial dose patterns to salivary glands for injury and recovery, dosimetric features of partitioned subvolumes were evaluated and the following key observations were made: (1) clinical and dosimetric features can be used to predict injury and recovery, i.e., damaged salivary glands are capable of regaining some of their function within 18 months post-RT, and the patterns for spatial dose distribution were different between injury vs. recovery, (2) for injury, dose within cOC, middle and inferior cPG was more important compared to dose to other regions; and in general, high dose to lower volume features was more important than the low-dose bath, and (3) for recovery, dose to cOC, superior-anterior and posterior cPG, middle-anterior cPG and superior-posterior iPG was more important compared to dose to other regions, and these regions tend to be the lower dose subvolumes in our study.

Compared to recovery, the critical subvolumes involved in injury were wider and more symmetric. This finding is consistent with our group’s voxel-based analysis results^28^. Since acinar cells are widely distributed in salivary glands, our observation is consistent with studies showing the injury of acinar cells is associated with the incidence of xerostomia^5–7^. For injury, we noticed that the superior portion of ipsilateral parotid gland is not as important as the superior and middle contralateral parotid gland. This may be due to the fact that dose to the ipsilateral parotid gland is generally high for HNC patients receiving RT, resulting in a lack of variation to detect the feature importance. Similarly, we may not have the ability to detect the importance of certain subvolumes for injury with the clinical datasets following existing treatment guidelines.

Nevertheless, the critical subvolumes involved in recovery centered on the superior iPG, superior and middle cPG, and cOC. Studies proposed that several theories for salivary regeneration, including progenitor cells reactivating^14,29,30^, and acinar cell self-duplication^31^. Recently, Emmerson *et al*. found that salivary glands regenerate after radiation injury through SOX2 - mediated secretory cell replacement, and parasympathetic nerves innveration is sufficient to maintain SOX2 expression in adult human salivary gland^32^. Since the main excretory duct of parotid gland located in superior and middle portion of the gland, we could posit that radiation injury to the main excretory duct at the first branch, and the hilum of gland, which contains the innervation and the blood supply, would cause lack of innervation, lack of perfusion, and potential for obstruction of the ducts. These changes might lead to loss of acinar cells and low expression of SOX2 in the adult salivary progenitor cells, and further loss of duct proliferation, leading to long-term xerostomia. Therefore, we speculated that superior iPG, superior and middle cPG regions identified in recovery analysis maybe consistent with this model. We further speculated that the similar mechanisms could possibly exist in the oral cavity which contains minor salivary glands. However, in this work, it is important to recognize that we used a geometrically constructed surrogate for the oral cavity and that our ability to make further conclusions regarding these observations are limited.

Interestingly, for injury, a smaller percentage of subvolumes receiving high dose was more important than low-dose bath, whereas recovery depends on typical low dose regions. Although speculative, an increased low-dose threshold in critical subvolumes where parasympathetic nerves and salivary progenitor cells reside in could provide less repair to the high dose area, thus decreasing the chance of recovering from xerostomia. This indicates that, clinically, there may be a greater potential for recovery if some portion of the glands are reduced to a low dose even lower than we currently consider in our treatment plans (for example, in our study, for superior-anterior portion of contralateral parotid, the median D50 recovery group is 940 cGy [range: 25–4295 cGy], while for non-recovery group is 1616 cGy [range: 226–5056 cGy]).

For recovery, it is noticeable that a few DVH features in the subvolumes indicate an increase in dose improves the chance of recovery. These features are in high dose subvolumes and suggest that an increase in dose here may offer the opportunity to decrease dose in the lower dose regions that have higher importance on recovery. However, we could not rule out the possibility that the coefficients representing an increase in dose to improve recovery are the result of noise or the relatively small sample size (N = 176) compared to the larger number of candidate DVH features as well as clinical features (p = 214).

Strengths of this study include the prospective xerostomia assessment collection which occurred at the point of care, generating a longitudinal history of radiation-induced xerostomia, our ability to segment the salivary glands and produce parameterized subvolumes, and the use of ridge logistic regression model to understand the relative importance of the dose features while controlling for the clinical variables. This study also has limitations. First, we acknowledged the limitation of the CTCAE scale, specifically, the low resolution and the subjectivity of the scale. However, we mitigated these limitations in the following ways: (1) individual patients were followed by the same attending physician across their care, (2) every attending followed the same criteria when assessing xerostomia, and (3) adaptation was not considered when assessing xerostomia recovery, e.g., clinicians considered grade 2 xerostomia if patients carry water with them all the time, while grade 1 xerostomia if patients no longer need water 24/7, but do have some dry mouth. We also lacked the variation of the magnitude of recovery, and did not have the ability to stratify patients and investigated on different recovery levels. Future research may be benefit on patient-reported xerostomia and objectively assessed xerostomia (salivary flow). Second, we could not rule out the possibility that our study may be subject to selection bias due to patients’ loss to follow-up after completion of RT. Third, we were not able to assess model performance in a separate test dataset due to our limited sample size, though the potential for overfitting was mitigated in our methodologic approach. Fourth, in our study, the minor salivary glands were represented by geometrically-derived oral cavity surrogate. The effect of the spatial dose distribution of minor salivary glands needs to be further studied. Last, the generalizability of our study remains limited as all the patients were from a single institution. External validation studies involving more patients and multiple institutions are needed.

In summary, we have demonstrated that the influences of spatial dose patterns in salivary glands for xerostomia injury and recovery were different. Compared to injury, recovery has increased importance to the subvolumes receiving lower dose. Within the subvolumes, injury tends to have increased importance towards D10 from D90. Further research on identifying the spatial dose patterns within oral cavity related to injury and recovery is needed. More work is needed on quantitatively comparing variability between dose features as they related to the derived hypotheses from the present study (e.g. the thresold of low dose to preserve recovery). Future validations are warranted to provide insights into applying selectively sparing strategies to treatment planning for injury prevention and recovery preservation.

## Supplementary information

Dose/Volume histogram patterns in Salivary Gland subvolumes influence xerostomia injury and recovery

## Data Availability

The datasets generated during and/or analyzed during the current study is publicly available, at 10.6084/m9.figshare.7250234.
