# Supplementary material for: Dose/Volume histogram patterns in Salivary Gland subvolumes influence xerostomia injury and recovery
Source: Sci Rep. 2019 Mar 5;9:3616. doi: 10.1038/s41598-019-40228-y (PMC6401158; doi:10.1038/s41598-019-40228-y)
Supplement: Supplementary file 1 — Dose/Volume histogram patterns in Salivary Gland subvolumes influence xerostomia injury and recovery [file 41598_2019_40228_MOESM1_ESM.docx]

**Title: Dose/Volume histogram patterns in Salivary Gland subvolumes influence xerostomia injury and recovery**

Peijin Han, MD, MHS*^1^, Pranav Lakshminarayanan, MS^1^, Wei Jiang, PhD^2^, Ilya Shpitser, PhD^3^, Xuan Hui, MD, ScM^4^, Sang Ho Lee, PhD^1^, Zhi Cheng, MD, MPH^1^, Yue Guo, MD, MHS^5^, Russell H. Taylor, PhD^3^, Sauleh A. Siddiqui, PhD^2^, Michael Bowers, MS^1^, Khadija Sheikh, PhD^1^, Ana Kiess, MD, PhD^1^, Brandi R. Page, MD^1^, Junghoon Lee, PhD^1^, Harry Quon, MD, MS^1^, Todd R. McNutt, PhD^1^

**Affiliation:** ^1^Department of Radiation Oncology and Molecular Radiation Sciences, Johns

Hopkins University, Baltimore, MD, USA; ^2^Department of Civil Engineering, Johns Hopkins System Institute, Johns Hopkins University, Baltimore MD, USA; ^3^Department of Computer Science, Johns Hopkins University, Baltimore, MD, USA; ^4^Department of Public Health Science, University of Chicago, Chicago, IL; ^5^Department of Epidemiology, Johns Hopkins Bloomberg School of Public Health, Baltimore, MD

| **Supplement Table 1. The Distribution of The Volume for Segmented zones, with 3mm Expansion** | | |
| --- | --- | --- |
| ROI of PG | Mean±Sd (cc) | Percentage (Mean±Sd, %) |
| Contralateral superior-medial | 3.5±1.4 | 6.1±2.0 |
| Contralateral superior-anterior | 6.4±2.1 | 11.0±2.1 |
| Contralateral superior-posterior | 6.5±2.3 | 11.1±2.4 |
| Contralateral middle-medial | 6.7±2.5 | 11.2±1.8 |
| Contralateral middle-anterior | 9.5±3.3 | 15.9±1.5 |
| Contralateral middle-posterior | 10.3±3.5 | 17.3±1.6 |
| Contralateral inferior-medial | 3.9±1.6 | 6.5±1.5 |
| Contralateral inferior-anterior | 5.8±2.2 | 9.8±1.9 |
| Contralateral inferior-posterior | 6.6±2.5 | 11.1±2.1 |
| Ipsilateral superior-medial | 3.5±1.4 | 6.2±2.0 |
| Ipsilateral superior-anterior | 6.4±2.2 | 11.1±2.0 |
| Ipsilateral superior-posterior | 6.5±2.4 | 11.2±2.2 |
| Ipsilateral middle-medial | 6.6±2.5 | 11.1±1.6 |
| Ipsilateral middle-anterior | 9.4±3.2 | 15.9±1.5 |
| Ipsilateral middle-posterior | 10.0±3.5 | 17.1±1.6 |
| Ipsilateral inferior-medial | 3.8±1.6 | 6.4±1.4 |
| Ipsilateral inferior-anterior | 5.8±2.2 | 9.9±1.8 |
| Ipsilateral inferior-posterior | 6.5±2.5 | 11.1±2.0 |

| **Supplement Table 2: The Coefficients of DVH Features, for Injury and Recovery** | | | | |
| --- | --- | --- | --- | --- |
| Feature | Coefficient (injury) | Importance (injury) | Coefficient (recovery) | Importance (recovery) |
| contraoc_d10 | 1.37E-03 | 86.75 | -1.90E-02 | -83.47 |
| contraoc_d20 | 1.39E-03 | 88.79 | -1.74E-02 | -76.21 |
| contraoc_d30 | 1.43E-03 | 91.72 | -1.50E-02 | -65.52 |
| contraoc_d40 | 1.46E-03 | 93.45 | -1.58E-02 | -69.23 |
| contraoc_d50 | 1.44E-03 | 92.14 | -1.51E-02 | -65.99 |
| contraoc_d60 | 1.43E-03 | 91.18 | -1.28E-02 | -55.99 |
| contraoc_d70 | 1.42E-03 | 90.83 | -7.96E-03 | -34.89 |
| contraoc_d80 | 1.40E-03 | 89.08 | -5.49E-03 | -24.05 |
| contraoc_d90 | 1.25E-03 | 78.33 | -9.11E-03 | -39.93 |
| ipsioc_d10 | 1.14E-03 | 70.03 | 4.88E-04 | 2.14 |
| ipsioc_d20 | 1.09E-03 | 66.09 | -4.41E-03 | -19.31 |
| ipsioc_d30 | 1.12E-03 | 68.20 | -5.55E-03 | -24.33 |
| ipsioc_d40 | 1.20E-03 | 74.34 | -1.07E-02 | -46.70 |
| ipsioc_d50 | 1.24E-03 | 77.01 | -1.87E-02 | -82.07 |
| ipsioc_d60 | 1.24E-03 | 77.65 | -1.92E-02 | -83.97 |
| ipsioc_d70 | 1.27E-03 | 79.37 | -1.75E-02 | -76.84 |
| ipsioc_d80 | 1.25E-03 | 78.44 | -1.35E-02 | -59.30 |
| ipsioc_d90 | 1.07E-03 | 65.08 | -6.28E-03 | -27.53 |
| pcre03z1s1d10 | 1.05E-03 | 63.52 | 6.61E-03 | 28.98 |
| pcre03z1s1d20 | 1.06E-03 | 63.90 | 6.32E-03 | 27.68 |
| pcre03z1s1d30 | 1.06E-03 | 64.01 | 5.68E-03 | 24.87 |
| pcre03z1s1d40 | 1.03E-03 | 61.53 | 5.18E-03 | 22.67 |
| pcre03z1s1d50 | 9.99E-04 | 59.59 | 3.13E-03 | 13.73 |
| pcre03z1s1d60 | 9.73E-04 | 57.65 | 1.24E-03 | 5.44 |
| pcre03z1s1d70 | 9.45E-04 | 55.60 | -3.06E-03 | -13.40 |
| pcre03z1s1d80 | 9.05E-04 | 52.64 | -7.00E-03 | -30.67 |
| pcre03z1s1d90 | 8.53E-04 | 48.81 | -1.07E-02 | -46.91 |
| pcre03z1s2d10 | 8.78E-04 | 50.62 | -1.74E-02 | -76.09 |
| pcre03z1s2d20 | 8.49E-04 | 48.47 | -1.88E-02 | -82.55 |
| pcre03z1s2d30 | 8.42E-04 | 48.01 | -2.06E-02 | -90.28 |
| pcre03z1s2d40 | 8.57E-04 | 49.08 | -2.04E-02 | -89.54 |
| pcre03z1s2d50 | 8.77E-04 | 50.56 | -2.00E-02 | -87.57 |
| pcre03z1s2d60 | 8.99E-04 | 52.17 | -1.78E-02 | -78.14 |
| pcre03z1s2d70 | 9.02E-04 | 52.40 | -1.51E-02 | -66.19 |
| pcre03z1s2d80 | 9.04E-04 | 52.60 | -1.22E-02 | -53.61 |
| pcre03z1s2d90 | 9.09E-04 | 52.97 | -9.50E-03 | -41.65 |
| pcre03z1s3d10 | 9.62E-04 | 56.87 | -8.00E-03 | -35.03 |
| pcre03z1s3d20 | 9.49E-04 | 55.88 | -1.22E-02 | -53.31 |
| pcre03z1s3d30 | 9.39E-04 | 55.15 | -1.50E-02 | -65.82 |
| pcre03z1s3d40 | 9.24E-04 | 54.06 | -1.38E-02 | -60.68 |
| pcre03z1s3d50 | 9.12E-04 | 53.16 | -1.40E-02 | -61.46 |
| pcre03z1s3d60 | 8.99E-04 | 52.16 | -1.37E-02 | -59.95 |
| pcre03z1s3d70 | 8.78E-04 | 50.66 | -1.30E-02 | -57.16 |
| pcre03z1s3d80 | 8.48E-04 | 48.40 | -1.28E-02 | -56.10 |
| pcre03z1s3d90 | 8.23E-04 | 46.61 | -9.08E-03 | -39.80 |
| pcre03z2s1d10 | 1.50E-03 | 96.51 | -1.25E-02 | -54.91 |
| pcre03z2s1d20 | 1.52E-03 | 97.72 | -9.36E-03 | -41.00 |
| pcre03z2s1d30 | 1.52E-03 | 98.07 | -4.89E-03 | -21.44 |
| pcre03z2s1d40 | 1.52E-03 | 98.13 | -1.91E-03 | -8.35 |
| pcre03z2s1d50 | 1.54E-03 | 99.17 | -2.85E-05 | -0.12 |
| pcre03z2s1d60 | 1.55E-03 | 100.00 | 1.38E-03 | 6.04 |
| pcre03z2s1d70 | 1.54E-03 | 99.18 | 3.39E-03 | 14.86 |
| pcre03z2s1d80 | 1.52E-03 | 97.67 | 6.63E-03 | 29.07 |
| pcre03z2s1d90 | 1.48E-03 | 95.34 | 3.39E-03 | 14.86 |
| pcre03z2s2d10 | 1.40E-03 | 88.94 | -1.69E-02 | -73.90 |
| pcre03z2s2d20 | 1.29E-03 | 81.34 | -2.04E-02 | -89.22 |
| pcre03z2s2d30 | 1.23E-03 | 76.29 | -1.84E-02 | -80.48 |
| pcre03z2s2d40 | 1.17E-03 | 72.40 | -1.53E-02 | -67.00 |
| pcre03z2s2d50 | 1.14E-03 | 69.94 | -1.54E-02 | -67.34 |
| pcre03z2s2d60 | 1.13E-03 | 69.48 | -1.44E-02 | -63.08 |
| pcre03z2s2d70 | 1.13E-03 | 68.89 | -1.45E-02 | -63.37 |
| pcre03z2s2d80 | 1.12E-03 | 68.34 | -1.55E-02 | -67.90 |
| pcre03z2s2d90 | 1.10E-03 | 66.93 | -1.66E-02 | -72.86 |
| pcre03z2s3d10 | 1.44E-03 | 92.17 | 8.72E-03 | 38.22 |
| pcre03z2s3d20 | 1.46E-03 | 93.28 | 7.68E-03 | 33.65 |
| pcre03z2s3d30 | 1.44E-03 | 92.08 | 4.50E-03 | 19.72 |
| pcre03z2s3d40 | 1.42E-03 | 90.86 | 1.28E-03 | 5.62 |
| pcre03z2s3d50 | 1.39E-03 | 88.71 | -2.63E-03 | -11.52 |
| pcre03z2s3d60 | 1.35E-03 | 85.23 | -3.83E-03 | -16.76 |
| pcre03z2s3d70 | 1.29E-03 | 81.05 | -5.49E-03 | -24.08 |
| pcre03z2s3d80 | 1.27E-03 | 79.23 | -4.97E-03 | -21.77 |
| pcre03z2s3d90 | 1.21E-03 | 74.90 | -5.29E-03 | -23.16 |
| pcre03z3s1d10 | 1.41E-03 | 89.91 | -9.84E-03 | -43.12 |
| pcre03z3s1d20 | 1.41E-03 | 89.91 | -8.28E-03 | -36.27 |
| pcre03z3s1d30 | 1.41E-03 | 89.70 | -6.96E-03 | -30.51 |
| pcre03z3s1d40 | 1.41E-03 | 89.55 | -5.46E-03 | -23.93 |
| pcre03z3s1d50 | 1.40E-03 | 89.35 | -3.06E-03 | -13.42 |
| pcre03z3s1d60 | 1.41E-03 | 90.15 | -1.74E-03 | -7.60 |
| pcre03z3s1d70 | 1.43E-03 | 91.21 | -8.22E-04 | -3.60 |
| pcre03z3s1d80 | 1.42E-03 | 90.82 | -7.61E-04 | -3.33 |
| pcre03z3s1d90 | 1.41E-03 | 89.89 | 2.51E-03 | 11.01 |
| pcre03z3s2d10 | 1.34E-03 | 84.93 | 3.73E-03 | 16.35 |
| pcre03z3s2d20 | 1.33E-03 | 83.82 | 5.99E-03 | 26.25 |
| pcre03z3s2d30 | 1.28E-03 | 80.54 | 8.18E-03 | 35.82 |
| pcre03z3s2d40 | 1.23E-03 | 76.91 | 9.09E-03 | 39.82 |
| pcre03z3s2d50 | 1.19E-03 | 73.40 | 9.19E-03 | 40.25 |
| pcre03z3s2d60 | 1.15E-03 | 70.72 | 8.92E-03 | 39.08 |
| pcre03z3s2d70 | 1.10E-03 | 66.74 | 1.00E-02 | 43.98 |
| pcre03z3s2d80 | 1.06E-03 | 63.75 | 1.16E-02 | 50.94 |
| pcre03z3s2d90 | 9.87E-04 | 58.67 | 1.24E-02 | 54.45 |
| pcre03z3s3d10 | 1.35E-03 | 85.17 | 1.22E-03 | 5.33 |
| pcre03z3s3d20 | 1.32E-03 | 83.34 | 2.91E-03 | 12.74 |
| pcre03z3s3d30 | 1.31E-03 | 82.78 | 3.70E-03 | 16.21 |
| pcre03z3s3d40 | 1.29E-03 | 81.40 | 3.98E-03 | 17.42 |
| pcre03z3s3d50 | 1.28E-03 | 80.42 | 3.72E-03 | 16.30 |
| pcre03z3s3d60 | 1.25E-03 | 78.44 | 3.00E-03 | 13.16 |
| pcre03z3s3d70 | 1.21E-03 | 74.98 | 2.01E-03 | 8.81 |
| pcre03z3s3d80 | 1.14E-03 | 69.75 | 9.35E-04 | 4.10 |
| pcre03z3s3d90 | 1.03E-03 | 61.86 | 2.63E-03 | 11.54 |
| pire03z1s1d10 | 6.87E-04 | 36.55 | -1.17E-02 | -51.42 |
| pire03z1s1d20 | 7.31E-04 | 39.76 | -1.08E-02 | -47.26 |
| pire03z1s1d30 | 7.49E-04 | 41.15 | -9.24E-03 | -40.47 |
| pire03z1s1d40 | 7.37E-04 | 40.27 | -8.84E-03 | -38.75 |
| pire03z1s1d50 | 6.99E-04 | 37.44 | -6.10E-03 | -26.74 |
| pire03z1s1d60 | 6.50E-04 | 33.81 | -7.34E-03 | -32.14 |
| pire03z1s1d70 | 5.60E-04 | 27.19 | -1.15E-02 | -50.29 |
| pire03z1s1d80 | 4.95E-04 | 22.38 | -8.91E-03 | -39.04 |
| pire03z1s1d90 | 4.01E-04 | 15.43 | -9.88E-03 | -43.28 |
| pire03z1s2d10 | 1.95E-04 | 0.22 | -1.35E-02 | -59.32 |
| pire03z1s2d20 | 2.03E-04 | 0.85 | -1.31E-02 | -57.62 |
| pire03z1s2d30 | 2.22E-04 | 2.20 | -1.16E-02 | -50.85 |
| pire03z1s2d40 | 2.24E-04 | 2.36 | -1.10E-02 | -48.37 |
| pire03z1s2d50 | 2.15E-04 | 1.70 | -9.98E-03 | -43.71 |
| pire03z1s2d60 | 2.05E-04 | 0.98 | -8.76E-03 | -38.38 |
| pire03z1s2d70 | 1.95E-04 | 0.20 | -7.47E-03 | -32.72 |
| pire03z1s2d80 | 1.92E-04 | 0.00 | -6.37E-03 | -27.89 |
| pire03z1s2d90 | 1.99E-04 | 0.52 | -4.81E-03 | -21.08 |
| pire03z1s3d10 | 4.64E-04 | 20.06 | -2.07E-02 | -90.66 |
| pire03z1s3d20 | 4.11E-04 | 16.17 | -2.28E-02 | -100.00 |
| pire03z1s3d30 | 3.74E-04 | 13.44 | -2.04E-02 | -89.37 |
| pire03z1s3d40 | 3.43E-04 | 11.12 | -1.74E-02 | -76.19 |
| pire03z1s3d50 | 3.20E-04 | 9.43 | -1.44E-02 | -63.10 |
| pire03z1s3d60 | 3.07E-04 | 8.46 | -1.18E-02 | -51.62 |
| pire03z1s3d70 | 2.89E-04 | 7.17 | -8.97E-03 | -39.30 |
| pire03z1s3d80 | 2.72E-04 | 5.88 | -5.32E-03 | -23.33 |
| pire03z1s3d90 | 2.49E-04 | 4.21 | -2.40E-03 | -10.50 |
| pire03z2s1d10 | 1.14E-03 | 69.92 | 8.55E-03 | 37.44 |
| pire03z2s1d20 | 1.13E-03 | 69.50 | 8.50E-03 | 37.24 |
| pire03z2s1d30 | 1.14E-03 | 69.74 | 6.85E-03 | 30.02 |
| pire03z2s1d40 | 1.11E-03 | 67.89 | 5.01E-03 | 21.97 |
| pire03z2s1d50 | 1.08E-03 | 65.58 | 4.57E-03 | 20.01 |
| pire03z2s1d60 | 1.05E-03 | 63.50 | 5.81E-03 | 25.44 |
| pire03z2s1d70 | 1.03E-03 | 61.80 | 7.14E-03 | 31.27 |
| pire03z2s1d80 | 1.03E-03 | 61.77 | 7.26E-03 | 31.80 |
| pire03z2s1d90 | 9.72E-04 | 57.58 | 7.32E-03 | 32.09 |
| pire03z2s2d10 | 7.42E-04 | 40.63 | 8.76E-03 | 38.38 |
| pire03z2s2d20 | 6.75E-04 | 35.65 | 5.13E-03 | 22.49 |
| pire03z2s2d30 | 6.36E-04 | 32.77 | 4.56E-03 | 19.97 |
| pire03z2s2d40 | 6.00E-04 | 30.11 | 2.45E-03 | 10.75 |
| pire03z2s2d50 | 5.60E-04 | 27.15 | 9.70E-04 | 4.25 |
| pire03z2s2d60 | 5.27E-04 | 24.71 | -1.18E-03 | -5.19 |
| pire03z2s2d70 | 4.91E-04 | 22.07 | -1.79E-03 | -7.86 |
| pire03z2s2d80 | 4.59E-04 | 19.70 | -3.00E-03 | -13.17 |
| pire03z2s2d90 | 4.05E-04 | 15.75 | -3.69E-03 | -16.16 |
| pire03z2s3d10 | 1.02E-03 | 61.14 | 2.90E-03 | 12.72 |
| pire03z2s3d20 | 9.09E-04 | 52.94 | -1.43E-03 | -6.28 |
| pire03z2s3d30 | 8.55E-04 | 48.94 | -1.12E-03 | -4.90 |
| pire03z2s3d40 | 8.12E-04 | 45.79 | 3.07E-04 | 1.34 |
| pire03z2s3d50 | 7.18E-04 | 38.87 | 1.78E-03 | 7.80 |
| pire03z2s3d60 | 6.53E-04 | 34.01 | 5.07E-04 | 2.22 |
| pire03z2s3d70 | 5.89E-04 | 29.32 | -8.02E-04 | -3.51 |
| pire03z2s3d80 | 5.34E-04 | 25.25 | -2.30E-03 | -10.06 |
| pire03z2s3d90 | 5.02E-04 | 22.87 | -2.34E-03 | -10.27 |
| pire03z3s1d10 | 1.21E-03 | 75.01 | 5.55E-03 | 24.33 |
| pire03z3s1d20 | 1.21E-03 | 75.49 | 3.49E-03 | 15.29 |
| pire03z3s1d30 | 1.23E-03 | 76.58 | 2.42E-03 | 10.60 |
| pire03z3s1d40 | 1.22E-03 | 75.55 | 2.68E-03 | 11.75 |
| pire03z3s1d50 | 1.22E-03 | 75.53 | 2.66E-03 | 11.64 |
| pire03z3s1d60 | 1.22E-03 | 75.90 | 2.90E-03 | 12.70 |
| pire03z3s1d70 | 1.23E-03 | 76.31 | 3.06E-03 | 13.42 |
| pire03z3s1d80 | 1.21E-03 | 75.45 | 3.82E-03 | 16.72 |
| pire03z3s1d90 | 1.18E-03 | 73.20 | 5.06E-03 | 22.18 |
| pire03z3s2d10 | 1.09E-03 | 66.42 | 4.72E-03 | 20.69 |
| pire03z3s2d20 | 1.03E-03 | 61.96 | 3.35E-03 | 14.68 |
| pire03z3s2d30 | 9.99E-04 | 59.55 | -1.59E-03 | -6.95 |
| pire03z3s2d40 | 9.54E-04 | 56.22 | -4.84E-03 | -21.19 |
| pire03z3s2d50 | 8.95E-04 | 51.93 | -6.47E-03 | -28.34 |
| pire03z3s2d60 | 8.50E-04 | 48.58 | -6.06E-03 | -26.56 |
| pire03z3s2d70 | 8.00E-04 | 44.90 | -4.12E-03 | -18.03 |
| pire03z3s2d80 | 7.46E-04 | 40.90 | -2.44E-03 | -10.71 |
| pire03z3s2d90 | 6.95E-04 | 37.16 | -1.10E-03 | -4.83 |
| pire03z3s3d10 | 1.14E-03 | 70.14 | -3.24E-04 | -1.42 |
| pire03z3s3d20 | 1.10E-03 | 67.35 | -1.85E-03 | -8.11 |
| pire03z3s3d30 | 1.04E-03 | 62.86 | -4.78E-03 | -20.95 |
| pire03z3s3d40 | 9.77E-04 | 57.98 | -4.84E-03 | -21.19 |
| pire03z3s3d50 | 9.04E-04 | 52.59 | -6.30E-03 | -27.59 |
| pire03z3s3d60 | 8.45E-04 | 48.17 | -7.17E-03 | -31.40 |
| pire03z3s3d70 | 7.90E-04 | 44.15 | -4.28E-03 | -18.76 |
| pire03z3s3d80 | 7.52E-04 | 41.38 | -5.17E-03 | -22.64 |
| pire03z3s3d90 | 6.73E-04 | 35.48 | -4.12E-03 | -18.05 |
| scre03d10 | 1.38E-03 | 87.80 | -6.61E-03 | -28.96 |
| scre03d20 | 1.35E-03 | 85.51 | -7.78E-03 | -34.10 |
| scre03d30 | 1.31E-03 | 82.80 | -9.04E-03 | -39.59 |
| scre03d40 | 1.29E-03 | 80.82 | -1.08E-02 | -47.12 |
| scre03d50 | 1.26E-03 | 79.04 | -1.20E-02 | -52.49 |
| scre03d60 | 1.23E-03 | 76.57 | -1.24E-02 | -54.35 |
| scre03d70 | 1.22E-03 | 75.96 | -1.25E-02 | -54.57 |
| scre03d80 | 1.22E-03 | 75.61 | -1.11E-02 | -48.71 |
| scre03d90 | 1.21E-03 | 75.08 | -1.09E-02 | -47.63 |
| sire03d10 | 1.09E-03 | 65.95 | -2.02E-04 | -0.88 |
| sire03d20 | 1.04E-03 | 62.62 | -8.64E-04 | -3.79 |
| sire03d30 | 1.01E-03 | 60.22 | -8.13E-04 | -3.56 |
| sire03d40 | 9.79E-04 | 58.09 | -1.81E-03 | -7.91 |
| sire03d50 | 9.70E-04 | 57.47 | -3.15E-03 | -13.79 |
| sire03d60 | 9.58E-04 | 56.54 | -3.79E-03 | -16.61 |
| sire03d70 | 9.20E-04 | 53.76 | -4.55E-03 | -19.94 |
| sire03d80 | 8.75E-04 | 50.40 | -5.06E-03 | -22.16 |
| sire03d90 | 8.77E-04 | 50.55 | -5.44E-03 | -23.84 |

^*^pi: ipsilateral parotid gland; Pc: contralateral parotid gland; si: ipsilateral submandibular gland; sc: contralateral submandibular gland. Re03: contour + 3mm expansion.


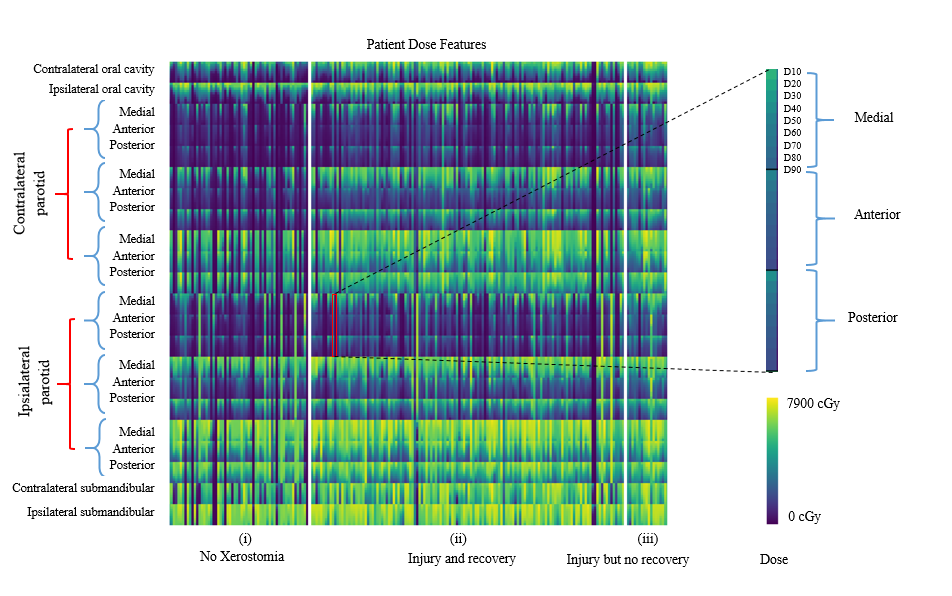


Figure Legend:

Supplement Figure 1. Dose distribution patterns for patients with (i) no xerostomia, (ii) injury followed by recovery, and (iii) injury but no recovery: a) dose features (darker blue indicates lower dose and brighter yellow indicates higher dose, range: 0-7900 cGy).
